# Use of photodynamic inactivation for *in vitro* reduction of prevalent bacteria in Fournier's Gangrene

**DOI:** 10.1590/S1677-5538.IBJU.2017.0312

**Published:** 2018

**Authors:** Nalisson Marques Pereira, Luciano Santos Feitosa, Ricardo Scarparo Navarro, Dora Inés Kozusny-Andreani, Naacia Marques Pereira Carvalho

**Affiliations:** 1 Departamento de Engenharia Biomédica, Universidade Brasil, São Paulo], SP, Brasil

**Keywords:** Fournier Gangrene, Methylene Blue, Clostridium perfringens

## Abstract

Fournier's Gangrene (FG) is an infectious disease caused by several synergic microbes, with high morbidity and mortality rates; therefore, the search for new less invasive and mutilating treatments, with faster recovery, has been proposed. Surgical intervention, the use of several systemic and topic antibiotics, and hyperbaric oxygen therapy are currently the best approach for the treatment of these patients. The use of Photodynamic Inactivation (PDI) aims to lower morbidity and mortality, by reducing bacterial microbiota and speeding wound healing. In the present study, viable bacteria were separated in four groups: Group L-/F- (no irradiation with red laser and absence of methylene blue photosensitizer), Group L-/F+ (no irradiation with red laser and presence of methylene blue), Group L+/F- (irradiation with red laser and absence of methylene blue) and L+/F+ (irradiation with red laser associated to methylene blue). In all groups, exposure time to treatment was 5, 10 and 15 minutes. The concentration of methylene blue photosensitizer was 0.1mg/L, and the dose of red laser (660nm wave length) was 176.9mW/cm^2^. Following irradiation, the reduction of number of bacteria was evaluated, and the results were expressed in colony forming units (CFU) and as exponential reduction. As the main results, in the L+/F+ group, there were no *Clostridium perfringens* and *Staphylococcus aureus* CFUs and there was a reduction of *Escherichia coli* that was not observed in the other groups.

## INTRODUCTION

Fournier's Gangrene (FG) is a polymicrobial infection caused by several aerobic and anaerobic microorganisms, that, synergistically, causes a necrotizing fasciitis, involving genitals, perineum and perianal region (1, 2). The most common microorganisms of FG are: gram positive aerobes, in special *Staphylococcus aureus, Staphylococcus epidermidis, Streptococcus viridans* and *Streptococcus faecalis*. Among anaerobes, the most common are *Bacteroides fragilis, Bacteroides melaninogenicus*, Gram positive coccus and *Clostridium sp. Among Gram* negative aerobes, it is observed the presence of *Escherichia coli, Klebsiella pneumoniae, Pseudomonas aeruginosa and Proteus mirabilis.* Synergistically, these bacteria act by different mechanisms and rapidly disseminates the infectious process (3).

Among microorganisms that cause FG, the most frequent isolated aerobes are: *Escherichia coli, Klebsiella pneumonie and Staphylococcus aureus*, and the most frequent anaerobes are *Bacteroides fragilis* and *Clostridium species* (4). Aside from bacteria, other microorganisms such as fungus, yeasts and virus may be present in FG and this fact is leading many researchers to search for alternative therapies to efficiently eliminate these pathogens. Photodynamic treatment (PDT) may represent an efficient treatment to eliminate these germs (5). Recent studies show that Photodynamic Inactivation (PDI) may be a viable alternative, since the action mode of photosensitizer (PS) over microbial cells is markedly different from the typical action of most antibiotic drugs (6).

PDI is based on topic or systemic administration of a non-toxic PS, followed by low dose irradiation of visible light with adequate wave length (7). This technique destroys target cells by means of oxidation that cause cellular lysis and inactivation of membrane proteins (8). In the presence of oxygen present in the cells, activated PS may react with neighbor molecules by means of transfer of electrons or hydrogen, producing free radicals (reaction type I) or transfer of energy to oxygen (reaction type II), producing oxygen singlet. Both ways cause cellular death (9, 10). Therefore, in view of the potential of this technique to treat bacterial infections, among other microorganisms, the objective of this work was to inactivate prevalent bacteria in FG, using PDI.

Among the most used photosensitizers (PS) in PDI, some stand out: natural occurring porphyrins, particular Protoporphyrin IX, chlorophylls and biocompatible PS that don't generate toxicity at dark (9). The most promising for human use for PDI are chlorines and phenothiazines: they absorb light with high wave length with adequate penetration in live tissues with low toxicity.

The use of PDI in medicine is becoming more relevant, since it is a reality in the treatment of diseases such as cancer, periodontitis, dermatologic diseases such as actinic keratosis and common acne, venereal diseases such as acuminate condyloma, among others (11-13).

Most studies in Urology using Photodynamic Therapy (PDT) are related to malign tumors such as bladder and prostate cancer (14, 15); our study aims to investigate the role of PDT in treating infectious diseases such as Fournier's Gangrene, one of the most morbid diseases in Urology.

## MATERIAL AND METHODS

### 

#### Bacterial Lines

It was used lines of *Escherichia coli* CCCD *(Coleção de Culturas Cefar Diagnóstica) Staphylococcus aureus* CCCD S003, (CEFAR Diagnóstica, Brasil), and *Clostridium perfringens* ATCC13124 (American Type Culture Collection). Culture media used included Tryptic Soy Agar (TSA, Oxoid^®^) supplemented with defibrinated goat blood (5%) for culture of S. *aureus* and C. *perfringens,* and for *E. coli* it was used agar eosin methylene blue (EMB, Oxoid^®^). Inoculums were prepared in Tryptic Soy Broth culture media (TSB, Oxoid^®^).

#### Preparation of methylene blue and red laser

PS methylene blue (*Fórmula e Ação,* São Paulo, SP, Brazil) was used at the concentration of 0.1 mg/mL for each sample. PS was dissolved in sterile double distilled water and filtered in a sterile membrane (Millipore, São Paulo, Brazil). Source of light used was red laser (Recover, MMOptics^®^, São Carlos, Brazil), with wavelength of 660 nm, energy density of 26.3 J/cm^2^, energy of 10 J, potency 100 mW. Periods of 5, 10 and 15 minutes of irradiation were used in an area of 0.56 cm^2^, that generated an irradiation of 176.9mW/cm^2^, according to Junqueira et al. protocol (16).

#### Preparation of bacterial samples and photosensitization

Bacterial lines were incubated in Tryptic Soy Broth culture media (TSB Oxoid^®^), for 24 hours at 37°C, *Escherichia coli and Staphylococcus aureus* in aerobic conditions and *Clostridium perfringens* in anaerobic environment. After that period, they were centrifuged at 4,000 rpm for 10 minutes; the supernatant was discharged and the pellet was resuspended in sterile solution of NaCl 0.5% and again centrifuged. This procedure was repeated five times. The obtained pellet was resuspended in 1mL of sterile NaCl 0.5% solution. Next, from a suspension of 10^6^ viable cells/mL of *Escherichia coli, Staphylococcus aureus* and *Clostridium perfringens,* 24 essays were performed, six for each experimental group. These essays were divided in 4 experimental groups: Group L-/F- (no irradiation with red laser and absence of methylene blue photosensitizer), Group L-/F+ (no irradiation with red laser and presence of methylene blue), Group L+/F- (irradiation with red laser and absence of methylene blue) and L+/F+ (irradiation with red laser associated to methylene blue). Microbial sample of group L-/F-included 1.0mL of bacterial suspension and 0.05mL of saline; microbial samples of groups L-/F+ and L+/F+ included 1.0mL of bacterial suspension and 0.05mL of methylene blue solution. After preparation, microorganisms remained for 15 minutes at 36°C incubated in methylene blue (0.1mg/mL) in dark environment and, next, they were irradiated with red laser. In every cycle of 5 minutes (in a total of 15 minutes), a sample of 0.1mL was removed and cultured in blood agar media for *S. aureus* and *C. perfringens* and EMB agar for *E. coli.* Bacterial suspension was evenly distributed with the aid of Drigalski sterile loops and the plates were incubated at 37°C for 24 hours. After that period, it was performed the counting of colonies to evaluate the photodynamic activity over bacterial lines. All procedures were performed in triplicates. Results were expressed in colony forming units (CFU). After final count of bacterial CFUs, final results were expressed in log and exponential regression.

### Statistical analysis

Data were statistically analyzed by analysis of variance (ANOVA) for totally randomized experiments, with statistical F calculus and their respective p-values. When p<0.05, media of treatments were compared using Tukey method, calculating the minimal significant difference for alpha (α)=0.05, using the software Graph Pad Prism 6.

## RESULTS

**Figure 1:**
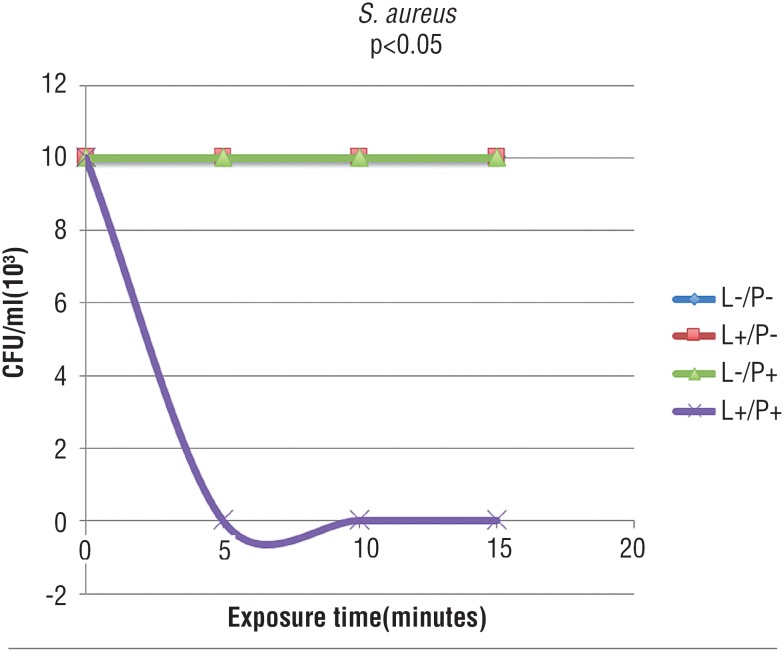
Mean CFU/mL of *S. aureus* submitted to the following treatments: saline as control (L-/P-), laser and saline (L+/P-), photosensitizer (L-/P+), and laser and photosensitizer (L+/P+) (Tukey test, p<0.05).

**Figure 2:**
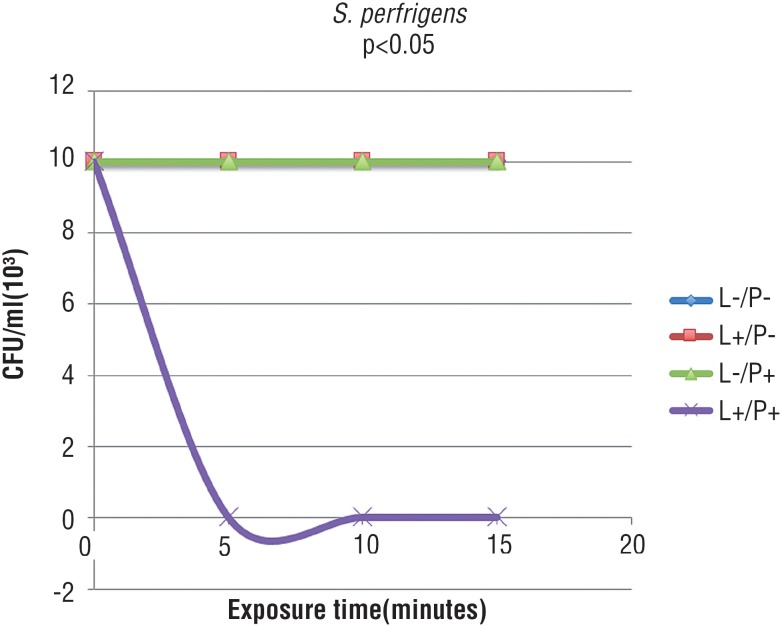
Mean CFu/mL of *C. perfrigens* submitted to the following treatments: saline as control (L-/P-), laser and saline (L+/P-), photosensitizer (L-/P+), and laser and photosensitizer (L+/P+) (Tukey test, p < 0.05).

**Figure 3:**
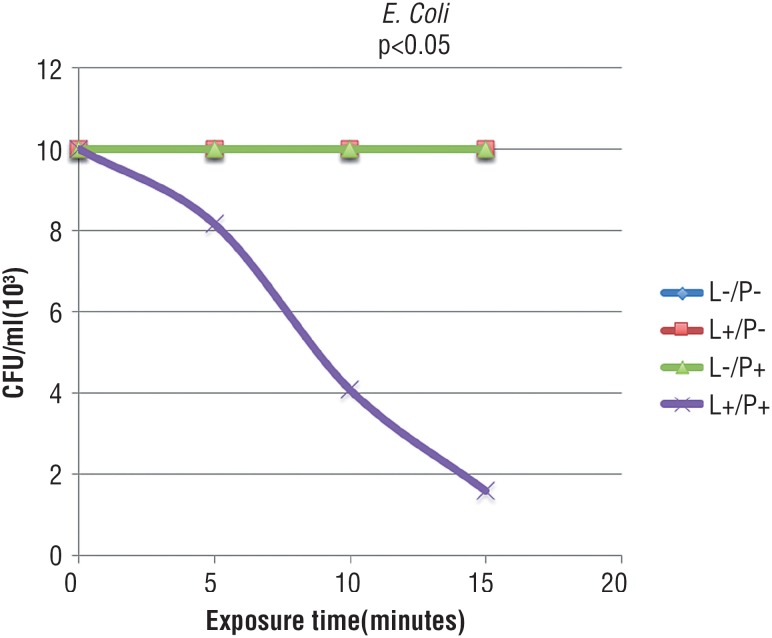
Mean CFU/mL of *E. coli* submitted to the following treatments: saline as control (L-/P-), laser and saline (L+/P-), photosensitizer (L-/P+), and laser and photosensitizer (L+/ P+). (Tukey test, p < 0.05).

Results obtained by statistical analysis of variance and Tukey test showed significant reduction of *Staphylococcus aureus*, *Clostridium perfringens* and *E. coli.* Figure-1 and Figure-2 show that, in the presence of laser and absence of PS, bacteria were not sensitive to phototherapy after 5, 10 and 15 minutes of exposure. As observed in the group treated only with methylene blue, there was no bacterial inactivation in all studied times, demonstrating that microorganisms were not also sensitive to PS alone. In Figures 1-3, graphic lines of control groups overlapped, since results were similar. In the group irradiated and with associated methylene blue, it was observed complete bacterial reduction regardless the increase of treatment time; after 5 minutes, all bacteria were inactivated.

In relation to *E. Coli,* Figure-3 shows exponential regression of the group treated with laser and methylene blue. This reduction was not time-dependent, since the increase of time to 10 or 15 minutes, lead to a reduction of 03log10. In the groups treated only with PS (L-F+) and in the groups treated only with laser, it was not observed exponential regression of microorganisms. As shown in Table-1, CFU media (log) of *S. aureus* and *C. perfrigens* bacteria were sensitive to PDI. *E. coli* species also showed sensitivity to treatment, however with exponential reduction.

**Table 1:** CFU log reduction of *S. aureus* and *C. perfrigens* and exponential reduction of *E. coli*.

Experimental groups						
	L-/F-	L+/F-	L-/F+	L+/F+(5)	L+/F+(10)	L+/F+(15*)
*E. coli*	10^6^	10^6^	10^6^	8.16×10^3^	4.08×10^3^	1.59×10^3^
*S. aureus*	10^6^	10^6^	10^6^	0	0	0
*C. perfrigens*	10^6^	10^6^	10^6^	0	0	0

* minutes

## DISCUSSION

This study showed that photodynamic inactivation associated to PS methylene blue was efficient to inactivate *Staphylococcus aureus, Clostridium perfringens* and *E. coli,* observed in FG (severe necrotizing fasciitis involving several bacterial species that need new treatments to lower the high rates of morbidity and mortality (1-3)).

The use of these bacteria is in accordance of the fact that in FG, there is multiple simultaneous presence of aerobic and anaerobic microorganisms; we used a sample of each category: a Gram positive aerobic, a Gram negative aerobic and an anaerobic bacteria. The choice of *Staphylococcus aureus, Clostridium perfringens* and *E. Coli* was based in the fact that in literature, they are referred as the most frequent in their category (4). It was not possible to perform a study with the three bacteria in the same culture conditions, since they are aerobic and anaerobic.

Although methylene blue is used widely due to its cytotoxicity against several microorganisms, such as bacteria and fungus (8, 17) this study is the first to show elimination of prevalent bacteria in FG. However, according to Kharkwal et al. (18) and Fuchs et al. (19), correct dose of PS and action of light source are very important in the elimination of microorganisms in PDI. Therefore, the concentration of PS was adjusted to 0.1 mg/mL in all studied groups: *S. aureus, C. perfringens and E. coli,* at this concentration, methylene blue is not cytotoxic when singly administrated. Our results show that the group treated with only methylene blue (L-/P+) showed microbial growth similar to that observed in the control group, without radiation and PS (L-F-). Similarly, when laser was applied singly (group L+/F-) the three different bacterial species grew similarly to control group. These data are consistent to the demonstration that methylene blue had no toxicity for bacterial cell. Also, it was evident that only light without the presence of PS was not able to promote microorganisms death.

The association of red laser and methylene blue showed complete inactivation of *S. aureus* and *C. perfrigens* when light was applied for 5 minutes in the presence of the PS, even when these bacteria were cultured along with *E. coli* after 24 hours of incubation. Several studies in literature corroborate this results, showing that 4 to 5 minutes of irradiation exposure time are sufficient to inactivate microorganisms (20, 21).

In relation to *E. coli,* there was no complete inactivation after 5 minutes of PDI, and even when exposure time was increased to 10 and 15 minutes. According to literature data, some factors may have influenced the non-complete elimination of bacteria, such as structural difference of the wall of Gram positive and Gram negative bacteria; the relative permeability of Gram positive membranes may be a facilitator of entrance of PS, and the more complex external membrane of Gram negative bacteria may work as a barrier to PS entrance. These data show that different bacteria, in special in relation to their different structures, make difficult a single therapy that can eliminate all kinds of bacteria (22, 23). This paper showed the efficiency of methylene blue as PS and of low potency red laser to inhibit the growth of all bacterial species studied. Therefore, it must be used as basis for *in vivo* studies.

## CONCLUSIONS

The use of low potency red laser associated to PS methylene blue was efficient to inactivate prevalent bacteria in Fournier's Gangrene.

It was observed complete inactivation of *S. aureus* and *Clostridium perfrigens* bacteria in treated groups, regardless the time of exposure. In relation to E. coli, there was no complete inactivation, but it was observed a statistically significant reduction of these microorganisms.
